# Matrix-Mediated Delivery of Silver Nanoparticles for Prevention of *Staphylococcus aureus* and *Pseudomonas aeruginosa* Biofilm Formation in Chronic Rhinosinusitis

**DOI:** 10.3390/pharmaceutics15102426

**Published:** 2023-10-05

**Authors:** Bhuvanesh Yathavan, Tanya Chhibber, Douglas Steinhauff, Abigail Pulsipher, Jeremiah A. Alt, Hamidreza Ghandehari, Paris Jafari

**Affiliations:** 1 Department of Molecular Pharmaceutics, University of Utah, Salt Lake City, UT 84112, USA; bhuvanesh.yathavan@utah.edu (B.Y.); tanya.chhibber@hci.utah.edu (T.C.); abigail.pulsipher@utah.edu (A.P.); jeremiah.alt@hsc.utah.edu (J.A.A.); hamid.ghandehari@pharm.utah.edu (H.G.); 2 Utah Center for Nanomedicine, University of Utah, Salt Lake City, UT 84112, USA; douglas.steinhauff@bms.com; 3 Department of Biomedical Engineering, University of Utah, Salt Lake City, UT 84112, USA; 4 Department of Otolaryngology—Head and Neck Surgery, University of Utah School of Medicine, Salt Lake City, UT 84132, USA; 5 Center for Integrative Genomics, University of Lausanne, 1015 Lausanne, Switzerland

**Keywords:** chronic rhinosinusitis, biofilm, in situ gel, sustained release, silver nanoparticle, SELPs

## Abstract

Chronic rhinosinusitis (CRS) is a chronic health condition affecting the sinonasal cavity. CRS-associated mucosal inflammation leads to sinonasal epithelial cell death and epithelial cell barrier disruption, which may result in recurrent bacterial infections and biofilm formation. For patients who fail medical management and elect endoscopic sinus surgery for disease control, bacterial biofilm formation is particularly detrimental, as it reduces the efficacy of surgical intervention. Effective treatments that prevent biofilm formation in post-operative patients in CRS are currently limited. To address this unmet need, we report the controlled release of silver nanoparticles (AgNps) with silk-elastinlike protein-based polymers (SELPs) to prevent bacterial biofilm formation in CRS. This polymeric network is liquid at room temperature and forms a hydrogel at body temperature, and is hence, capable of conforming to the sinonasal cavity upon administration. SELP hydrogels demonstrated sustained AgNp and silver ion release for the studied period of three days, potent in vitro antibacterial activity against *Pseudomonas aeruginosa* (**** *p* < 0.0001) and *Staphylococcus aureus* (**** *p* < 0.0001), two of the most commonly virulent bacterial strains observed in patients with post-operative CRS, and high cytocompatibility with human nasal epithelial cells. Antibacterial controlled release platform shows promise for treating patients suffering from prolonged sinonasal cavity infections due to biofilms.

## 1. Introduction

Chronic rhinosinusitis (CRS) is a chronic health condition that affects the paranasal sinuses. CRS impacts approximately 5% of the global population and 11.5% of adults in the United States [1]. Patients with CRS typically exhibit two or more sinonasal symptoms, such as nasal congestion, nasal discharge, facial pain, and a loss of smell for at least 12 weeks, leading to significant declines in their quality of life [2,3]. In addition to these sinonasal symptoms, individuals with CRS often present with comorbidities, including asthma, depression, migraines, cognitive deficits, and sleep dysfunction [4,5,6,7]. The sinonasal mucosa of CRS patients is characterized by the presence of epithelial layer damage, edema, and infiltration of immune cells [8]. Initial treatment for CRS primarily involves medical management, which includes saline irrigation, topical intranasal corticosteroid sprays, and, depending on the clinical phenotype, antibiotics and/or oral steroids [2,3]. However, these therapies provide limited symptomatic relief to recalcitrant CRS patients [2,3,9]. For patients who do not experience significant improvement with medical management, endoscopic sinus surgery (ESS) is often considered as a secondary intervention to achieve better outcomes [10,11]. For these patients, the presence of bacterial biofilms can worsen post-surgical outcomes, leading to the persistence of symptoms, mucosal inflammation, and recurrent infections [12]. Notably, within six months following ESS, biofilms have been shown to significantly deteriorate the patients’ quality of life [12]. Bacterial biofilms are clusters of polymicrobial bacteria that adhere to surfaces and are embedded within a self-produced extracellular matrix. Bacteria residing within a biofilm network exhibit remarkable resilience, being up to 1,000 times more resistant to antibiotic treatments than their planktonic counterparts [13,14]. Moreover, biofilms effectively evade the host’s defense mechanisms while simultaneously triggering the innate and adaptive immune systems, resulting in persistent inflammation [15,16]. Various bacterial species have been identified in the biofilm formed in CRS patients. Among those, *Staphylococcus aureus* (*S. aureus*) and *Pseudomonas aeruginosa* (*P. aeruginosa*) biofilms are most commonly observed and directly influential in shaping the severity of CRS [13,17,18,19,20,21].

ESS has been shown to disrupt the biofilm cycle and can prevent its formation in patients for up to three months post-surgery. However, the presence of residual bacteria in the sinonasal mucosa originating from previous bacterial biofilms contributes to unfavorable post-operative outcomes [22,23]. Despite the urgent clinical need, there is currently no approved or effective treatment to prevent the recurrence of bacterial biofilm formation in post-operative CRS patients [3]. While systemic and topical antibiotics are periodically administered to CRS patients and efficiently control symptomatic infections, they do not completely eradicate bacterial biofilms, leading to resistance and relapse [24,25]. Various strategies have been employed to neutralize and disrupt biofilms in these patients, including nasal saline irrigations, surfactants, probiotics, antimicrobial photodynamic therapy, pulsed ultrasound, and hydrodynamic flushing. However, these approaches have shown only partial effectiveness [14,26]. Thus, there is a crucial need for therapeutic strategies that can inhibit biofilm growth while avoiding the development of bacterial resistance, ultimately reducing the high revision surgery rates, which stand at 36.8% for patients with CRS [27].

Silver and, more recently, silver nanoparticles (AgNps) offer several bactericidal mechanisms that distinguish them from antibiotics. These mechanisms have demonstrated their effectiveness against a wide range of antibiotic-resistant bacteria, with a low probability of bacterial resistance development [28,29,30,31,32]. AgNps have been tested in sheep [33] and rabbit [34] models of CRS *S. aureus* biofilm, and both models showed complete elimination of biofilm [34]. However, when applied in clinical settings, colloidal silver and AgNps, delivered through sprays and nasal irrigation, did not exhibit the same level of efficacy [35,36]. The short residence time of AgNps in the nasal cavity is a key factor contributing to the lack of efficacy of these formulations in the clinic. To address this issue and enhance the effectiveness of antibacterial therapy, it is crucial to implement controlled delivery methods that extend the residence time of AgNps.

In situ forming thermoresponsive hydrogel networks offer a promising solution for the localized and controlled delivery of AgNps within the sinonasal cavity. These hydrogels exist in a liquid state at lower temperatures and transform into a three-dimensional polymeric network at body temperature, providing an opportunity for controlled therapeutic release in the sinonasal cavity [37,38,39]. These systems have already demonstrated their potential in preclinical models of CRS, successfully delivering corticosteroids and sustaining a reduction in inflammation for up to 4 weeks [40]. One notable class of hydrogels, known as silk-elastinlike protein polymers (SELPs), are genetically engineered recombinant protein-based polymers with thermoresponsive properties [41,42]. SELP hydrogels have been used for controlled release of therapeutics in the local environment [43,44,45,46,47]. SELPs combine the properties of silk-like (GAGAGS) and elastin-like (GVGVP) motifs. Through precise linear polymer sequence design and concentration adjustments, various physicochemical properties of SELPs, such as temperature-sensitivity, gel formation kinetics, rheology, pore size, and release rates, can be effectively controlled [41]. SELP-815K is a SELP analog with six repeats of a monomer that contains 8 units of silk, 15 units of elastin, and 1 unit of elastin substituted with lysine in a specific sequence [41]. Among different analogs, the specific sequence of silk and elastin units enables SELP-815K to act as a thermoresponsive polymer that is liquid at room temperature and forms a hydrogel at body temperature [41,43,44,45,46,48].

Hyaluronic acid (HA), a non-sulfated glycosaminoglycan and a major component of the extracellular matrix, has demonstrated its effectiveness in wound healing, mucosal regeneration, anti-inflammatory actions, and adhesion prevention following sinus surgeries in patients with CRS [49,50]. Consequently, incorporating HA into therapeutic formulations for CRS holds the potential to reduce inflammation and enhance wound healing in the sinonasal cavity, especially after ESS.

We aimed to incorporate AgNps into the SELP-815K matrix, thereby prolonging its residence time and achieve sustained AgNp release for effectively preventing biofilm formation in patients with CRS. We also incorporated HA in our formulation in order to confer wound healing and mild anti-inflammatory properties to our formulation. The in situ gelling property of SELP-815K can enable the physician to apply this novel antibacterial dressing directly to the sinonasal cavity via a catheter, where the dressing can then conform to the anatomical shape of the sinus cavity, forming a homogeneous coating. Herein, we report the development and full in vitro characterization of our novel antibacterial dressing containing AgNps loaded in SELP-815K delivery matrix. We show the cytocompatibility, bactericidal, and biofilm prevention properties, in vitro AgNp release kinetics, and rheological properties of the novel antibacterial dressings.

## 2. Materials and Methods

### 2.1. Material

*P. aeruginosa* (ATCC 27853), *S. aureus* (ATCC 25923), and human leukemia monocytic cell lines (THP-1 cells) were purchased from ATCC (Manassas, VA, USA). AgNps were purchased from nanoComposix (San Diego, CA, USA). HA was purchased from Lifecore Biomedical (Chaska, MN, USA). Lipopolysaccharide (LPS) from *Escherichia coli* O111:B4 and antibiotics were purchased from Sigma Aldrich (St. Louis, MO, USA). Human nasal epithelial cells (HNEpC) were purchased from Celprogen, Inc. (Torrance, CA, USA). SELP-815K (MDPVVLQRRDWENPGVTQLVRLAAHPPFASDPM-[GAGS(GAGAGS)_2_(GVGVP)_4_GKGVP(GVGVP)_11_(GAGAGS)_5_GA]_6_-GAMDPGRYQDLRSHHHHHH) was produced via fermentation, purified, and sheared, as previously described [41,48,51].

### 2.2. Characterization of AgNps

AgNps obtained from nanoComposix were coated with poly(vinyl pyrrolidone) (PVP) and dispersed in distilled water at a 5 mg/mL concentration. The surface plasmon resonance of AgNps was measured using UV-Vis spectrophotometry to confirm storage stability. The AgNps stock solution was sterile-filtered by passing is through a 0.2-µm filter (VWR, Radnor, PA, USA). The AgNps stock was diluted to 1:600 in deionized water, and absorbance was measured between 300 to 700 nm in 1-nm increments. The size and size distribution of AgNps were measured by transmission electron microscopy (TEM). Samples were drop-casted on TEM grids and analyzed using a JEOL JEM-1400 Plus Transmission Electron Microscope (Peabody, MA, USA). The hydrodynamic diameter and charge of AgNps were measured in 10 mM NaCl using dynamic light scattering in Malvern zeta-sizer Nano-ZS (Malvern, Worcestershire, UK). The concentration of Ag^+^ ions was quantified using inductively coupled plasma mass spectroscopy (ICP/MS) (Agilent 8900, Santa Clara, CA, USA) at the ICP/MS lab, Department of Geology and Geophysics, University of Utah. In brief, about 0.1 mL of AgNps was weighed in a polypropylene tube and diluted with 0.3 mL of water. Then, 0.6 mL of concentrated nitric acid was added, and once the reaction was complete, the solution was diluted to 10 mL with water. An external calibration curve was prepared from a 1g/L standard solution for silver (Inorganic Ventures, Christiansburg, VA, USA) with concentrations of 1, 2, 5, 10, and 20 ng/mL for Ag^+^. Diluted samples, calibration solutions, and blanks were added to 10 ng/mL Indium as the internal standard and quantified by ICP/MS. Trace metal grade pure chemicals were used for these experiments. The experiment was conducted in triplicate.

### 2.3. Minimum Inhibitory Concentration (MIC) and Minimum Bactericidal Concentration (MBC) Assays

The MIC and MBC concentrations of AgNps were determined using a macro dilution method [52]. Two mL of tryptic soy broth (TSB) containing different concentrations of AgNps were prepared in 5 mL culture tubes and inoculated with an overnight culture of *P. aeruginosa* or *S. aureus* adjusted to approximately 5 × 10^5^ colony forming units (CFU)/mL. The culture tubes were incubated at 37 °C for 24 h at 225 rpm. TSB was used as a positive control, and TSB containing ampicillin and polymyxin B were used as negative controls for bacterial growth. After 24 h incubation, visual observations were recorded, and the culture tubes were then serially diluted and plated on agar plates to determine the CFU/mL. MIC was determined as the lowest AgNp concentration in which no visual bacterial growth was observed. MBC was recorded as the lowest AgNp concentration in which a 3-log reduction of bacterial CFU occurred from the initial inoculum [53]. Biological replicates (*N* = 2) and technical replicates (*n* = 3) for each experiment were performed.

### 2.4. HA Concentration Optimization

The concentration of HA was optimized based on the concentration of cytokines released from THP-1 cells after activation with lipopolysaccharide (LPS). THP-1 cells were seeded in 24-well plates at a density of 450,000 cells per well. Phorbol 12-myristate 13-acetate (50 ng/mL) was used to differentiate THP-1 cells into macrophages. After two days of resting, the cells were exposed to LPS (1 µg/mL) and different concentrations of HA (0.5, 1, 2, 5, and 10 mg/mL) for 24 h. Sterile HA was used in all experiments. The supernatant was collected, and cytokine release (IL-6 and IL-8) was determined using an ELISA kit (Biolegend, San Diego, CA, USA).

### 2.5. Cytocompatibility Assessment—Cell Counting Kit-8 (CCK) Assay

HNEpCs were cultured on 6.5-mm diameter transwell plates with a 0.4-µm pore membrane in 24-well plates. Approximately 0.2 mL of media was added to the apical side and 0.6 mL media was added to the basal side. Culture media were replaced every day until 80% confluency was achieved. At 80% confluency, media on the basal side were replaced with fresh media. Media from the apical side were removed and 10 µL of either (1) SELP-815K (11%), (2) SELP-815K (11%) with HA (0.2%) (SELP/HA), (3) SELP-815K (11%) with HA (0.2%) and AgNps (SELP/HA/AgNps) (125, 625, and 1250 µg/mL), or (4) AgNps (125, 625, and 1250 µg/mL) was added, and the plates were incubated in a cell culture incubator for 10 min. Then, 0.2 mL of media was added to the apical side and incubated in a cell culture incubator for 24 h. Cells with no treatment were used as positive control, and 10% DMSO was used as negative control. After incubation, the cells were washed twice with cell culture media and viability was determined by comparing the metabolic activity of treated and non-treated cells using the cell-counting kit-8 (CCK) assay (Dojindo, Rockville, MD, USA), according to vendor’s instructions. Biological replicates (*N* = 2) and technical replicates (*n* = 3) for each experiment were performed.

### 2.6. Antibacterial Effect Assessment—Viable Count Assay

The antibacterial effect of the entire formulation was determined using a viable count assay. SELP/HA and SELP/HA/AgNps (625 µg/mL) were freshly prepared, added to 96-well plates, and incubated at 37 °C for 30 min to gel. Then, 100 µL of overnight *S. aureus* and *P. aeruginosa* cultures were adjusted to 5 × 10^5^ CFU/mL with fresh media, added to each well, and incubated in a humidified chamber at 37 °C for 24 h under static conditions. Wells without any treatment and ciprofloxacin (50 µg/mL) treatment were used as negative and positive controls, respectively. After incubation, 10 μL of sample from each well was serially diluted and plated to determine the viable CFU/mL. The log reduction in the bacterial count was determined by manually counting the colonies. Biological replicates (*N* = 2) and technical replicates (*n* = 3) for each experiment were performed.

### 2.7. Biofilm Prevention Assay—Colony Biofilm Assay

The biofilm prevention property of the entire formulation was determined using colony biofilm assay, as outlined by Merritt et al. [54], with slight modification. The concentration of overnight bacterial cultures of *S. aureus* and *P. aeruginosa* strains were adjusted to 5 × 10^5^ CFU/mL. Then, 20 µL of adjusted bacterial cultures were added to a 100 nm polycarbonate membrane placed on an agar plate. The bacterial culture was allowed to dry. SELP/HA, SELP/HA/AgNps (625 µg/mL), and ciprofloxacin (50 µg/mL) were freshly prepared, added to the dried bacterial culture, and the plates were incubated at 37 °C for 24 h. Polycarbonate filters without any treatment and ciprofloxacin (50 µg/mL) treatment were used as negative and positive controls, respectively. After incubation, the bacterial biofilm was removed from the polycarbonate membrane by vortexing (1 min), sonicating (5 min), and vortexing (30 s) in 10 mL of Lauria–Bertani broth media. The bacterial cultures were further serially diluted, and the CFU/mL from each polycarbonate membrane was measured using the plate spread method. The log reduction in the bacterial count was determined by manually counting the colonies. Separate polycarbonate filters with different treatments were prepared to analyze the biofilm formation through scanning electron microscopy (SEM), as described in Section 2.9. Biological replicates (*N* = 2) and technical replicates (*n* = 3) for each experiment were performed.

### 2.8. In Vitro Release Study

The release rate of Ag^+^ ions from the formulation was determined using a previously established method [45], with slight modification. Briefly, the appropriate amount of AgNps and HA was previously mixed in PBS and then added to SELP-815K and mixed well. The formulation was then loaded into 1 mL tuberculin syringe and allowed to gel overnight at 37 °C in a humid environment. The tip of the syringe was then cut, and cylindrical pieces of formulation were sectioned, accounting for approximately 30 mg by weight. The sectioned formulation was added to 24-well plates, and 2 mL of simulated nasal fluid (SNF) was added to each well. At various time points (1, 3, 6, 12, 24, 48, and 72 h), complete release media were collected and replaced with 2 mL of fresh release media. The collected release media from different time points were digested with nitric acid, and Ag^+^ content was measured using ICP/MS as described in Section 2.2. The influence of SNF on AgNps was studied through UV-Vis spectroscopy and TEM analysis. These experiments were conducted in triplicate.

### 2.9. Scanning Electron Microscopy (SEM)

Secondary electron images of the hydrogel were collected using a Quanta SEM system (Thermofisher FEI; Hillsboro, OR, USA). A beam energy of 15 kV and a spot size of 4 was used. Beam energy and current were optimized for high-resolution imaging. Samples were mounted on an aluminum stub using double-sided carbon tape. Prior to imaging, samples were coated with 20 nm Au/Pd using a Leica EM ACE 600 (Leica, Deerfield, IL, USA) to minimize sample charging. Hydrogels were directly coated with Au/Pd and analyzed to determine biofilm formation. Hydrogel sample preparation to study the interaction of AgNps was prepared, similar to previously described method [46].

### 2.10. Rheological Properties

The rheological properties of the formulations were determined using Malvern Kinexus Ultra+ Rheometer (Malvern Panalytical, Malvern, United Kingdom) equipped with cone and plate geometry with a 2° angle and 20 mm diameter. Viscosity was determined at different temperatures, applying a temperature ramp from 4 °C to 34 °C (5 °C/min) using an oscillatory procedure at an angular frequency of 6.283 rad/s. Following viscosity, the storage (G’) and loss (G”) moduli were measured using oscillatory sweep at 34 °C, strain 0.01%, and angular frequency of 6.283 rad/s. Formulations were freshly prepared before each run. Humidity was maintained with the help of an environmental chamber. The experiments were conducted in triplicate.

### 2.11. Statistical Analysis

Statistical analyses were performed using GraphPad Prism version 9.5.0 (GraphPad Software, San Diego, California, USA). Statistical comparisons were conducted using a one-way analysis of variance followed by Tukey’s multiple comparison tests to identify significant differences between the control and treatment groups. A *p*-value of less than 0.05 was considered significant. All data were represented as the mean ± standard deviation.

## 3. Results

### 3.1. AgNps Characterization

**Figure 1:**
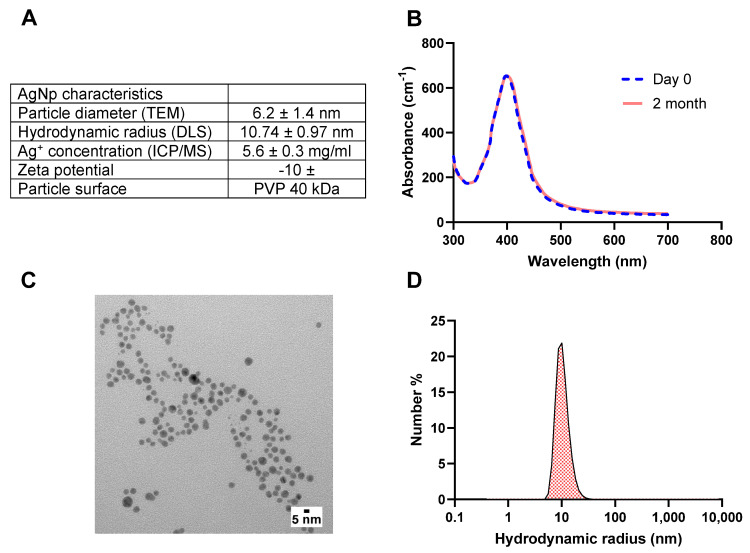
(**A**) AgNps features after characterization with TEM, DLS, spectrophotometry, and ICP/MS. (**B**) UV−Vis surface plasmon resonance spectrum indicates that AgNPs are colloidally stable upon receipt from the manufacturer and after 2 months in storage at 4 °C, (**C**) representative TEM images of AgNps, and (**D**) DLS size distribution demonstrate an average hydrodynamic radius of 10 nm for AgNps. TEM images were produced by nanocomposix. silver nanoparticles (AgNps), dynamic light scattering (DLS), transmission electron microscopy (TEM), and inductively coupled plasma mass spectroscopy (ICP/MS).

The AgNps characteristics measured via TEM, DLS, ICP/MS, and spectrophotometry are outlined in Figure 1A. The surface plasmon resonance of AgNps measured at different time points indicated that AgNps are stable in deionized water with no indication of aggregation nor reduction in AgNp concentration during storage (Figure 1B). The particle diameter measured via TEM was 6.2 ± 1.4 nm (Figure 1A), and the representative TEM image is shown in Figure 1C. As expected, the size of the particles obtained via DLS was slightly higher than the TEM measurement, as DLS measures the hydrodynamic radius (Figure 1A,D), which is influenced by PVP coating.

### 3.2. Identification of MIC and MBC of AgNps against S. aureus and P. aeruginosa

A macro dilution method was used to determine the MIC and MBC of AgNps against *S. aureus and P. aeruginosa*, which are listed in Table 1. The MIC for both strains was 7.8 µg/mL, the lowest concentration tested. The MBC of *P. aeruginosa* was lower than that of the MBC obtained for *S. aureus* (Table 2). Thus, the highest MBC concentration obtained (125 µg/mL) was selected for formulation development. For the sustained release formulation, two higher concentrations (625 µg/mL and 1250 µg/mL), corresponding to 5X and 10X of MBC, were also included for further optimization.

### 3.3. Optimization of HA Concentration for Anti-Inflammatory Effect

**Figure 2:**
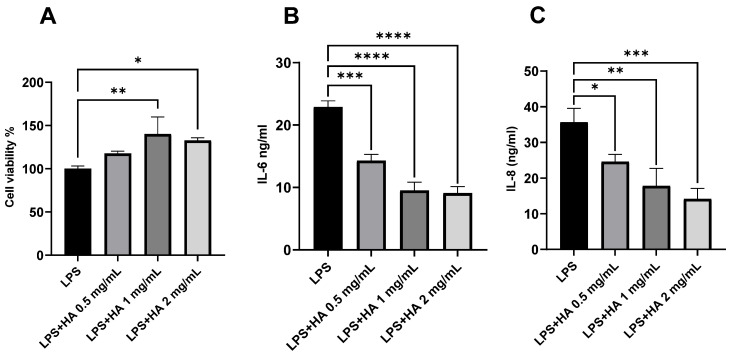
Effect of HA on the viability and pro-inflammatory cytokine release from LPS-stimulated THP-1 cells. HA significantly increased the (**A**) cell viability and decreased the secretion of (**B**) IL-6 and (**C**) IL-8. *n* = 3. Data are represented as mean ± SD. (* *p <* 0.05; ** *p <* 0.01; *** *p <* 0.001; **** *p <* 0.0001). Lipopolysaccharide (LPS), hyaluronic acid (HA).

Macrophages play a vital role in contributing to the pathogenesis and disease severity in CRS [55,56]. The minimum concentration of HA for inhibiting LPS-induced secretion of interleukin 6 (IL-6) and 8 (IL-8) from THP-1 cells was determined. LPS, a gram-negative bacterial cell wall component and an agonist to toll-like receptor 4, was used to trigger THP-1 cells to release inflammatory cytokines [54]. The concentration of LPS to trigger THP-1 cells without causing substantial cell death (cell viability > 70%) was selected for further study (1 µg/mL) [55]. HA (0.5, 1.0, 2.0 mg/mL) was added to LPS-stimulated THP-1 cells and significantly increased cell viability (Figure 2A). Furthermore, HA significantly reduced the secretion of IL-6 and IL-8 from LPS-stimulated THP-1 cells (Figure 2B,C) in a dose-dependent manner, and 2 mg/mL of HA was selected for further formulation development.

### 3.4. Cytocompatibility of Free AgNps and Hydrogel Formulations

**Figure 3:**
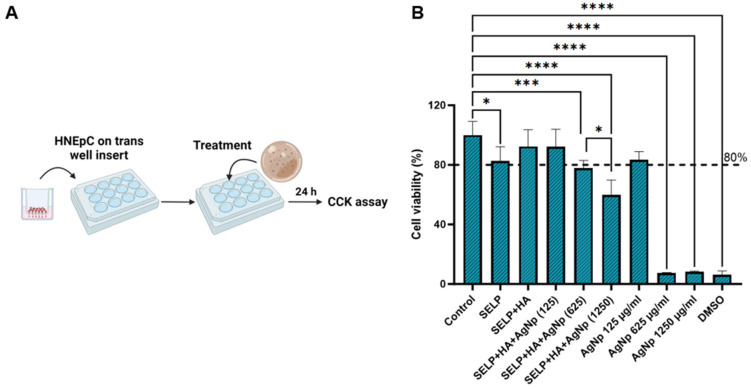
The cytocompatibility of hydrogel formulations in the presence of HNEpCs. (**A**) A schematic illustrating the cell viability experimental setup; (**B**) the percentage of viable cells after exposure to different free AgNps and hydrogel formulations compared to non-treated control cells, measured using the CCK-8 cell viability assay. Biological replicates (*N* = 2) and technical replicates (*n* = 3) for each experiment were performed. Data are represented as the mean ± SD. (* *p <* 0.05; *** *p <* 0.001; **** *p <* 0.0001). Human nasal epithelial cells (HNEpCs), cell counting kit-8 (CCK-8), silk-elastinlike protein polymers (SELPs), hyaluronic acid (HA), silver nanoparticles (AgNps). Numbers in parentheses are micrograms per mL.

The cytocompatibility of free AgNps and hydrogel formulation with HNEpCs was determined using the CCK-8 assay (Figure 3A). The free form of AgNps showed cytocompatibility only at 125 µg/mL concentration (cell viability 83.28 ± 5.63%) (Figure 3B). At 625 µg/mL and 1250 µg/mL, free AgNps reduced the HNEpCs’ viability to 7.36 ± 0.37% and 8.21 ± 0.45%, respectively (Figure 3B). The addition of SELP/HA and SELP/HA/AgNps (125 µg/mL) did not affect HNEpCs’ viability. Formulations containing 1250 µg/mL AgNps, significantly decreased HNEpCs’ viability (59.76 ± 10.05%; *p* = 0.0215), and formulations containing 625 µg/mL AgNps showed a 77.89 ± 5.12% (*p* = 0.008) viability compared to that of non-treated control cells (Figure 3B). According to the International Organization for Standardization (ISO 1099-5:2009), more than 70% cell viability is considered non-toxic for topical gel formulations [57]. Thus, a SELP/HA/AgNps formulation of 625 µg/mL was selected for further development, and the formulation containing 1250 µg/mL AgNps was excluded from the study.

### 3.5. Antibacterial Effect of Hydrogel Formulations Measured by Viable Count Assay

**Figure 4:**
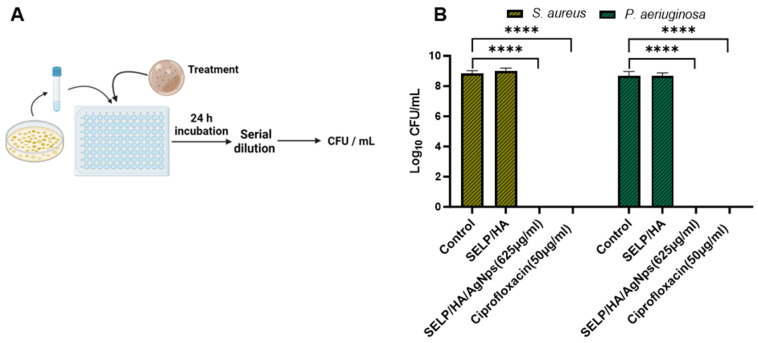
SELP/HA/AgNPs (625 µg/mL) showed a bactericidal effect against *S. aureus and P. aeruginosa*. (**A**) Schematic illustration of the cell viability assay. (**B**) The antibacterial effect is represented as log CFU/mL reduction in bacterial counts by treatment with the hydrogels compared to wide-spectrum antibiotic ciprofloxacin. Biological replicates (*N* = 2) and technical replicates (*n* = 3) for each experiment were performed. Data are represented as the mean ± SD. (**** *p <* 0.0001). Colony forming units (CFU), silk-elastinlike protein polymers (SELPs), hyaluronic acid (HA), silver nanoparticles (AgNps).

A viable count assay was conducted to determine the bactericidal effect of the hydrogel formulation against planktonic bacteria (Figure 4A). The viable *S. aureus* count for control and SELP/HA treatment was 7.43 × 10^8^ ± 3.28 × 10^8^ CFU/mL and 1.09 × 10^9^ ± 5.23 × 10^8^ CFU/mL. Similarly, the viable *P. aeruginosa* count for control and SELP/HA treatment for was 6.00 × 10^8^ ± 4.77 × 10^8^ CFU/mL and 5.09 × 10^8^ ± 2.79 × 10^8^ CFU/mL. The hydrogel formulation containing AgNps showed a complete bactericidal effect against *S. aureus* and *P. aeruginosa*, being comparable to ciprofloxacin, which was used as a positive control for antibacterial effect (Figure 4B). The hydrogel formulation without AgNps did not show a reduction in CFU/mL of bacteria, indicating that the bactericidal effect was due to the AgNps incorporated into the hydrogel matrix.

### 3.6. Biofilm Prevention Assessment with Matrix-Mediated Delivery of AgNps

**Figure 5:**
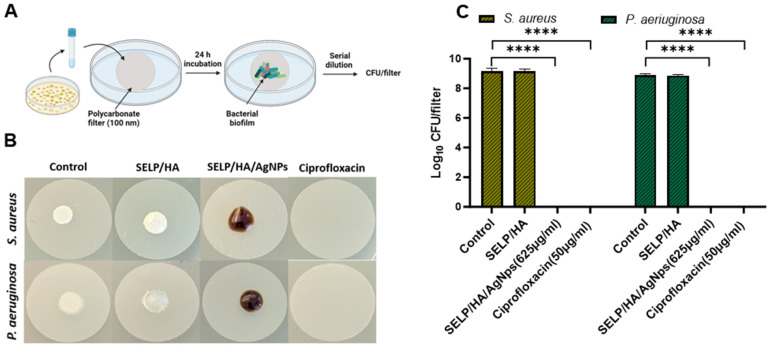
SELP/HA/AgNps (625 µg/mL) significantly prevented biofilm formation. (**A**) A schematic illustration of the colony biofilm experiment setup. (**B**) Images of polycarbonate filters after 24 h incubation with different treatments. The dark brown color observed in SELP/HA/AgNps (625 µg/mL) is due to the incorporation of AgNps. Notably, the AgNps stock solution is naturally dark in color. (**C**) Influence on CFU/filter due to treatment with different formulations. Biological replicates (*N* = 2) and technical replicates (*n* = 3) for each experiment were performed. Data are represented as the mean ± SD (**** *p <* 0.0001). Colony forming units (CFUs), silk-elastinlike protein polymers (SELPs), hyaluronic acid (HA), silver nanoparticles (AgNps), scanning electron microscopy (SEM).

**Figure 6:**
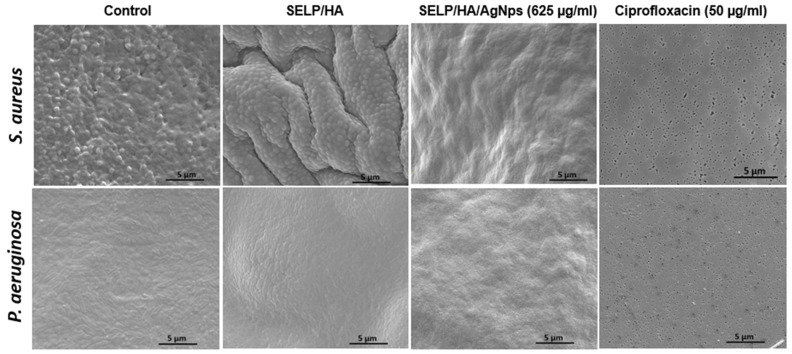
SEM images of polycarbonate filters showing the presence of biofilm in control and SELP/HA treatment. The treatment with SELP/HA/AgNps and ciprofloxacin showed complete biofilm prevention after a 24-h incubation. Silk-elastinlike protein polymers (SELPs), hyaluronic acid (HA), silver nanoparticles (AgNps), scanning electron microscopy (SEM).

The feasibility of the hydrogel formulation to prevent biofilm formulation was determined using a colony biofilm assay (Figure 5A) [54]. The negative control, with no treatment, showed a thin film of bacterial growth that could be visualized with the naked eye after 24 h incubation. The hydrogel formulation without AgNps showed bacterial growth on the surface of the hydrogel even though the gel covered the entire bacterial surface, indicating that the presence of gel alone did not hinder bacterial biofilm formation. The treatment with SELP/HA/AgNps (625 µg/mL) as well as ciprofloxacin inhibited biofilm formation (Figure 5B). The log CFU calculated for each filter showed that hydrogels incorporating AgNps significantly prevented biofilm formation with *S. aureus* (*p* < 0.0001) and *P. aeruginosa* (*p* < 0.0001), similar to the positive control for antibiofilm activity (Figure 5C). The polycarbonate filter analyzed by SEM confirmed the formation of biofilm, which was evident from the higher density of bacteria, the fusion of individual bacteria, and the presence of extracellular matrix observed in the high-magnification SEM image (Figure 6) [58]. The SEM image shows the presence of bacterial biofilm in control and SELP/HA treatments and the absence of biofilm and bacterial growth in SELP/HA/AgNps (625 µg/mL) and ciprofloxacin treatments (Figure 6).

### 3.7. Assessment of AgNps Release from Hydrogels

**Figure 7:**
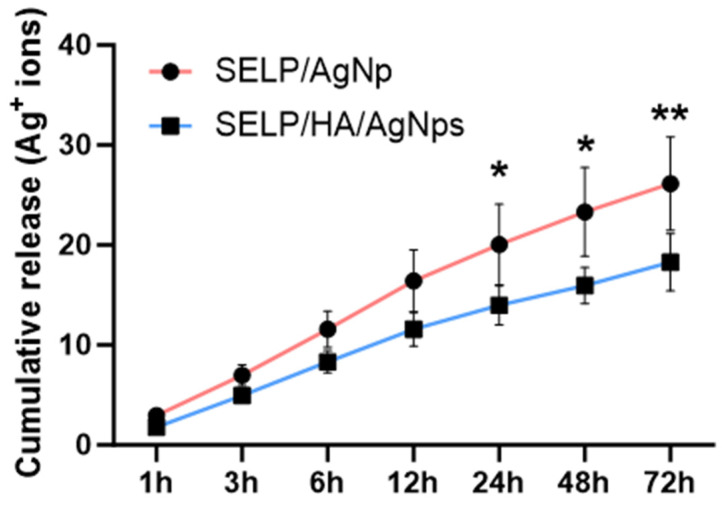
Release kinetics of Ag+ ions from SELP/AgNps (625 µg/mL) and SELP/HA/AgNps (625 µg/mL) in simulated nasal fluid. (* *p <* 0.05; ** *p <* 0.01). Silk-elastinlike protein polymers (SELPs), hyaluronic acid (HA), silver nanoparticles (AgNps).

**Figure 8:**
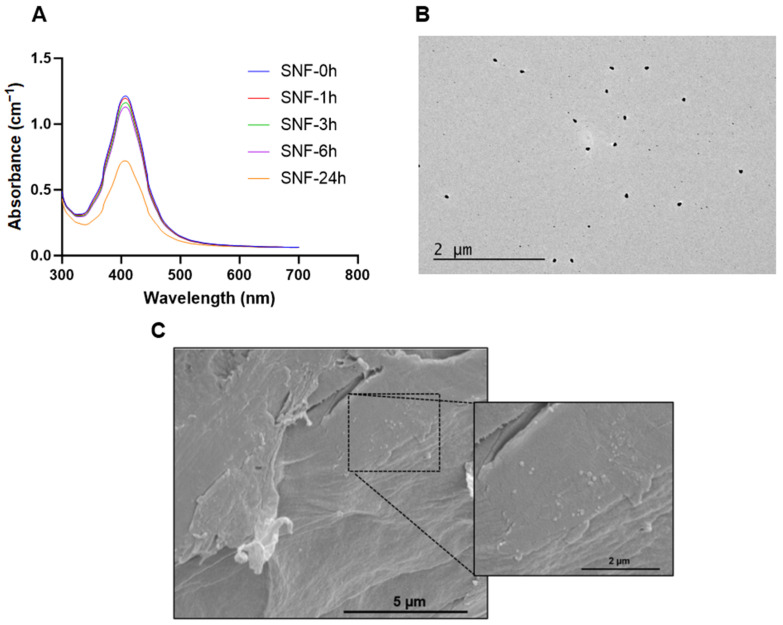
Changes to AgNps in the presence of SNF. (**A**) Surface plasmon resonance spectrum of AgNps after incubation in SNF, (**B**) TEM image of AgNp after 24 h incubation in SNF, (**C**) SEM image of SELP/HA/AgNps (625 µg/mL) after 24 h incubation in SNF. Silver nanoparticle (AgNps), simulated nasal fluid (SNF), silk-elastinlike protein polymers (SELPs), hyaluronic acid (HA), transmission electron microscopy (TEM), scanning electron microscopy (SEM).

The release kinetics of Ag^+^ ions play a crucial role in achieving sustained bactericidal effects. The kinetics of Ag^+^ ion release from SELP/AgNps (625 µg/mL) and the selected hydrogel formulation, SELP/HA/AgNps (625 µg/mL), was measured by in vitro release study in SNF. The cumulative release for both formulations at 6 h was 11.58 ± 1.82% and 8.31 ± 1.10% (Figure 7), indicating the absence of burst release. The AgNps and Ag^+^ ion release from both formulations showed sustained linear release kinetics, and the cumulative release until 12 h was not significantly different. Starting from 24 h, the release of AgNps and Ag^+^ ions significantly differed, indicating HA’s influence on release kinetics. The cumulative release achieved at 72 h for SELP/AgNps (625 µg/mL) and SELP/HA/AgNps (625 µg/mL) was 26.16 ± 4.68% and 18.29 ± 2.86%, respectively (Figure 7). The release of Ag^+^ ions from AgNps was minimal during the first six hours of incubation in SNF (Figure 8A). The particle size increased to 23.52 ± 19.06 nm (particles counted, *n* = 75) after 24 h of SNF incubation, indicating particle aggregation and precipitation (Figure 8B). The SEM analysis of SELP/HA/AgNps (625 µg/mL) hydrogel after 24 h of incubation in SNF showed nanoparticle interaction with the SELPs backbone (Figure 8B), leading to sustained release of Ag^+^ ions.

### 3.8. Rheological Characterization of the Final Antibacterial Dressing

**Figure 9:**
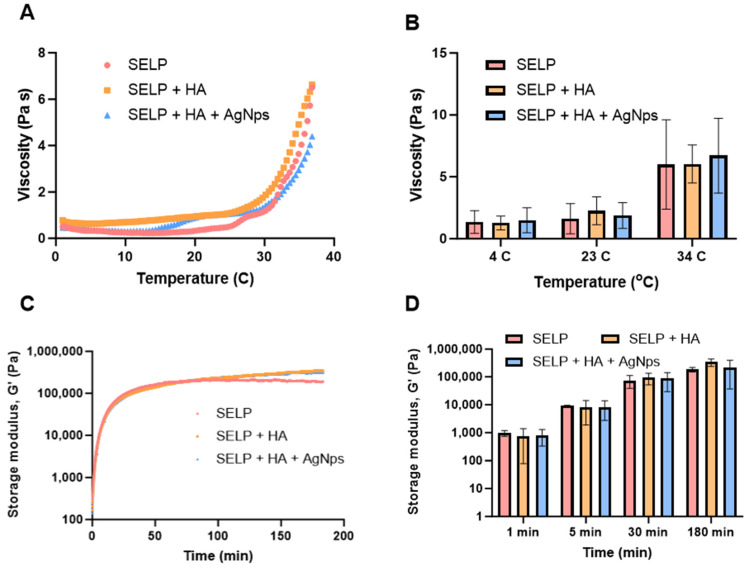
The thermoresponsive properties of SELP/HA/AgNPs (625 µg/mL). (**A**) Viscosity as a function of temperature, (**B**) viscosity at different time points, (**C**) storage modulus as a function of time, (**D**) storage modulus at different time points.

The rheological properties are crucial in determining the injectability and gelation kinetics of the antibacterial dressing. Therefore, we studied the rheological properties of three different formulations, SELPs, SELP/HA, and SELP/HA/AgNps (625 µg/mL). The injectability and gel formation of the formulations were assessed by measuring the viscosity as a function of temperature (Figure 9A). Viscosity was measured at 4, 23, and 34 °C to represent refrigerated, ambient, and nasal temperatures, respectively. The increase in viscosity indicated the initiation of gelation and the thermoresponsive property of the three formulations as a function of temperature (Figure 9A,B). The viscosity of the formulation did not change significantly with the addition of HA (2 mg/mL) and HA/AgNps (625 µg/mL) at different temperatures. The viscosity of the SELP/HA/AgNps (625 µg/mL) increased from (1.02 ± 0.86 Pa s) to (1.31 ± 0.41 Pa s) to (5.01 ± 0.87 Pa s) as the temperature increased from 4 °C to 23 °C to 34 °C. The trend was similar for formulation with SELPs and SELP/HA. The observed increase in the viscosity as a function of temperature was similar to the previous trend observed with SELP-815K [43,45]. The viscosity of the formulation at 4 °C and 23 °C would allow it to be injected through an applicator tip (larger than 18-gauge needle) used to access different sinuses in the clinic during ESS [59,60].

The sol-to-gel transition of the formulations was monitored by measuring storage modulus (G’) and loss modulus (G’’) over 3 h via an oscillatory time sweep at 34 °C. The storage modulus of the formulation SELP/HA/AgNps (625 µg/mL) was 8.29 kPa at 5 min, 86.87 kPa at 30 min, and 218.63 kPa at 180 min. A continuous increase in G’ until 180 min indicated the formation of silk–silk interactions (Figure 9C,D). The gelation point is defined as the time when G’ crosses G’’ during the oscillatory sweep cycle. The gelation time for SELPs, SELP/HA, and SELP/HA/AgNps (625 µg/mL) was less than 60 s after reaching 34 °C. The incorporation of HA or AgNps did not significantly alter the gelation time. Thus, the inclusion of HA and AgNPs did not significantly affect the gelation kinetics and the final gel properties, as measured by the rheological assessment.

## 4. Discussion

Bacterial biofilms can lead to poor outcomes of ESS in CRS patients [12]. Biofilm-positive patients with CRS undergo more revision surgeries than patients without biofilm-colonized sinuses, with biofilms present in 75% of patients undergoing revision surgeries [22]. Although ESS effectively removes and disrupts biofilms, it leaves behind residual planktonic bacteria, which can develop into new biofilms, diminishing the efficacy of surgery [23]. Oral antibiotics have limited penetration into the sinonasal mucosa [61] and current topical antibacterial treatments have failed to show efficacy, due to poor residence time [62]. Currently, no treatment prevents biofilm formation in CRS patients. There is an urgent need to develop therapeutics that prevent biofilm formation in patients with CRS that can be delivered following ESS to improve outcomes.

Herein, we developed an antibacterial dressing that prevents the formation of biofilms by sustained release of AgNps, which are non-antibiotic antibacterial agents. The sinonasal cavity of patients with CRS who undergo ESS is inherently inflamed and primed. Hence, the selection of formulation components with a minimal inflammatory response is cardinal. The formulation comprises SELP, recombinant protein polymers, and HA, an extracellular matrix component. SELP hydrogels have been used for sustained drug and gene delivery with no observable toxicity [43,44,45,63]. Aqueous solutions of SELPs can be easily applied through a catheter with the ability to conform to the anatomical shape of the sinonasal cavity to form a hydrogel.

The concentration of AgNps used in the polymeric solutions was optimized by a macro dilution method. In this study, the MBC obtained for AgNps with an average diameter of around 5 nm was higher for *S. aureus* than for *P. aeruginosa*. Gram-negative bacteria contain a thin peptidoglycan layer and are more susceptible to AgNps than gram-positive bacteria equipped with a thick peptidoglycan layer [28,64]. The biofilm-prevention property of three concentrations of AgNps, 125 µg/mL, 625 µg/mL, and 1250 µg/mL, corresponding to 1X, 5X, and 10X MBC, was tested using crystal violet assay. Even the lowest concentration of AgNps significantly reduced the biofilm formation, compared to non-treated controls (Supplement Figure S1). The mechanism by which AgNps attains the bactericidal effect is through the combination of the effects observed from AgNps and released Ag^+^ ions. AgNps can cause bacterial cell-wall destabilization (resulting in cellular content leakage and death), penetrate the cytoplasm and interact with cell organelles (thus affecting ATP synthesis and DNA replication), and generate reactive oxygen species (that induce oxidative stress) [28,29,30,31]. Similarly, Ag^+^ ions have also been shown to disrupt bacterial cell walls, damage cellular components, and cause oxidative stress [28,29,30,31]. AgNps are also known for disrupting quorum sensing, an active mechanism in understanding the environment and communicating within the bacterial species [65], thus directly affecting biofilm formation [66]. AgNps demonstrate efficacy against a broad spectrum of pathogenic bacteria, and the development of resistant bacterial strains to AgNps is rare compared to antibiotics, due to its multi-bactericidal mechanism [32].

The direct contact method was employed to determine the cytocompatibility of the entire antibacterial dressing with HNEpCs. Notably, only hydrogels containing high concentrations of AgNps (1250 µg/mL) showed toxicity to HNEpCs (Figure 3). In contrast, those with lower AgNps concentrations, still exhibiting high antibacterial and antibiofilm effects, remained at the accepted range of cytocompatibility for topical formulations. Importantly, the AgNps delivered through the hydrogel formulations demonstrated greater cytocompatibility than free AgNps (Figure 3B). This improved compatibility was primarily attributed to the slow release of AgNps or Ag+ ions from the hydrogel matrix. Therefore, higher concentrations of AgNPs can be delivered via hydrogels without causing significant inflammation or HNEpCs’ death. Formulations containing 625 µg/mL concentration of AgNps displayed acceptable cytocompatibility [57]. The viable count assay showed that the finalized formulation containing SELP/HA/AgNps (625 µg/mL) eliminated bacterial growth of both strains (*S. aureus* and *P. aeruginosa;* Figure 4). SELP/HA composition had similar bacterial counts to those of the negative control, indicating that the observed bactericidal effect was due to AgNps and not influenced by SELPs or HA.

In vitro models closely mimicking in vivo conditions, such as the colony biofilm assay, are valuable for optimizing formulation efficacy. Unlike other biofilm assays, in colony biofilm assay, the bacteria are grown on a polycarbonate filter and the nutrients are supplied from the agar below, similar to the in vivo condition in the sinonasal cavity. Biofilm formation in the absence of treatment was confirmed by SEM analysis (Figure 6). Treatment with the SELP/HA/AgNps (625 µg/mL) significantly prevented biofilm formation of both strains, mainly due to the sustained release of AgNps/Ag^+^ ions, which enhances the efficacy and reduces the toxicity associated with AgNps and Ag^+^ ions. The free form of AgNps (625 µg/mL) also demonstrated biofilm prevention potential (Supplement Figure S2); however, free AgNps are toxic to HNEpCs at this concentration (Figure 3B). Thus, our antibacterial dressing has an effective bactericidal effect against the tested planktonic bacteria, as well as biofilm prevention properties against bacteria attached to a substrate.

AgNps in other sustained-release formulations have shown bactericidal effects against various bacterial and multi-bacterial strains [67,68,69]. In CRS, biofilms are formed by single and polymicrobial bacterial strains [17], and the developed SELP/HA/AgNp antibacterial dressing can effectively control biofilm formation by multiple pathogenic bacterial species. The sustained release properties of our antibacterial dressing were evident from the in vitro release kinetics. The release study showed linear kinetics with a maximum of 18.29 ± 2.86% Ag^+^ ions released at 72 h from our final formulation. Based on previous studies with SELP hydrogels, the release kinetics of hydrophilic drugs from SELPs hydrogel is governed by diffusion through the pores, and the complete release of drugs occurs within 24 h [43,45]. The release kinetics of hydrophobic drugs is dictated by drug–drug and drug–polymer interactions and showed a sustained release for more than a week [46].

We hypothesize that the sustained Ag^+^ ion released from our formulation was due to AgNp-SELP-815K interactions, as demonstrated using SEM (Figure 8C). It is possible that the PVP-coated AgNps might interact with SELP-815K through electrostatic interactions and hydrogen bonding based on the structure of PVP [70]. The Ag^+^ ion released from AgNPs readily reacts with chloride present in SNF and forms AgCl precipitant, which, in turn, might interact with the hydrophobic domains of SELP-815K. The release of Ag^+^ ions from AgNps was demonstrated by the plasmon resonance spectrum of AgNps (Figure 8A), whereas precipitant formation was evident from TEM analysis (Figure 8B). The combination of these interactions was likely responsible for the sustained release of AgNPs, Ag^+^ ions, and AgCl. The influence of HA on release kinetics was minimal (Figure 7). In the in vivo and clinical setting, the delayed release of AgNps, Ag^+^ ions, and AgCl may result in the prolonged efficacy of ESS with substantially low toxicity associated with AgNps and Ag^+^ ions. More detailed studies are warranted to better understand the mechanisms of AgNps’ interactions with the SELPs backbone and HA vis-a-vis the controlled release profile observed.

Rheological properties play a crucial role in the injectability and bioaccumulation of our antibacterial dressing in the sinonasal cavity. The viscosity of all three formulations increased as a function of temperature, which denoted the thermoresponsive nature of SELP-815K. At 4 °C and 23 °C, the formulations exhibited lower viscosities, suggesting flow-like properties. As the temperature was raised to 34 °C, viscosity increased, indicating the initiation of the gelling process (Figure 9B). The addition of HA and AgNps did not significantly alter the thermoresponsive property of SELPs, but it did result in a slight reduction in viscosities, possibly due to a decrease in inter-silk interactions within SELP-815K [43,45]. Nevertheless, the viscosities of all three formulations permit an easy administration at 23 °C (room temperature) through an 18 G catheter that is typically used in the operation room during sinus surgeries [59].

The current products in the market for the treatment of CRS fail, mainly, due to variable distribution and low residence time within the sinonasal cavity. The low viscosity observed at the initial stages of gel formation ensures the ease of administration and spreading in the sinonasal cavity. The continuous increase in viscosity at 34 °C enables the antibacterial dressing to remain in the sinonasal cavity, prolonging residence time and enabling sustained AgNps release. In a clinical setting, we envision applying aqueous SELPs solutions after ESS, when the sinuses are open. Immediately after ESS, the mucociliary clearance would be significantly reduced due to inflammation and damage to the sinonasal mucosa, enabling the dressing to reside in the sinonasal cavity for an extended period. Thus, the developed systems can be applied to the sinonasal cavity of patients with CRS via a catheter for sustained release of AgNps to prolong the efficacy of the ESS through prevention of biofilm formation. These systems can also be used for other applications in which bacterial biofilms pose a challenge, such as burn wounds and chronic wounds [71].

We recognize several limitations in our study that should be considered while interpreting these results. The bactericidal effects were obtained in single bacterial species rather than in multi-bacterial species. For future work, the system should be tested with other resistant, patient-derived, and multi-bacterial species. These systems can also be used for biofilm disruption, which was not covered in this study. They can be further optimized to improve the release kinetics by altering the SELPs and HA concentrations. In the future, other potential non-antibiotic antibacterial agents, such as antimicrobial peptides and dendrimers, can be included in our hydrogel matrix. The therapeutic effect of HA, such as wound healing and mucosal regeneration, should be studied when applying this formulation in an in vivo model. Detailed analysis of free HA release and its potential interactions with the hydrogel network and silver nanoparticles needs to be conducted. Despite these limitations, our proof-of-concept study demonstrated the potential to provide a conforming injectable matrix for antibacterial and anti-inflammatory drug delivery for more effective and less-toxic treatment of CRS.

## 5. Conclusions

In situ hydrogels provide a unique advantage in preventing bacterial biofilm in patients with CRS by overcoming the shortcomings in the current clinical products. A SELPs hydrogel containing HA and AgNps was developed and characterized in vitro for its bactericidal effects against *S. aureus* and *P. aeruginosa.* This antibacterial dressing demonstrated appropriate thermoresponsive properties to enable uniform distribution and sustained release of AgNps in the sinonasal cavity. The sustained release of AgNps can potentially be translated to use in the clinic, ultimately improving post-operative outcomes for patients with CRS.

## Figures and Tables

**Table 1:** MIC and MBC for *S. aureus* and *P. aeruginosa*.

	MIC (µg/mL)	MBC (µg/mL)
*S. aureus* (gram +ve)	7.8	125
*P. aeruginosa* (gram −ve)	7.8	7.8

**Table 2:** MBC streak plate method and CFU/mL.

	*S. aureus*		*P. aeruginosa*	
	MBC (Streak Plate Method)	MBC (CFU/mL)	MBC (Streak Plate Method)	MBC (CFU/mL)
	Presence of Growth	Growth Reduction (%)	Presence of Growth	Growth Reduction (%)
Control	+	<99.9	+	<99.9
Polymyxin B (50 µg/mL)	+	<99.9	-	>99.9
Ampicillin (50 µg/mL)	-	>99.9	+	<99.9
AgNps (250 µg/mL)	-	>99.9	-	>99.9
AgNps (125 µg/mL)	-	>99.9	-	>99.9
AgNps (62.5 µg/mL)	+	<99.9	-	>99.9
AgNps (31.3 µg/mL)	+	<99.9	-	>99.9
AgNps (15.6 µg/mL)	+	<99.9	-	>99.9
AgNps (7.8 µg/mL)	+	<99.9	-	>99.9

Cells highlighted with green correspond to the absence of growth, confirming antibacterial activity, whereas red corresponds to bacterial growth.

## Data Availability

Data are contained within the article and supplementary material.

## Supplementary Materials

The following supporting information can be downloaded at: https://www.mdpi.com/article/10.3390/pharmaceutics15102426/s1, Figure S1. Biofilm disruption assessments with silver nanoparticles (AgNp) and antibiotic controls using the crystal violet assay; Figure S2. Agar plates showing the inhibition of bacterial growth; Figure S3. *Staphylococcus aureus* colony biofilm assessments after treatment with different antibacterial formulations; Figure S4. *Pseudomonas aeruginosa* colony biofilm assessments after treatment with different antibacterial formulations; Figure S5. Scanning electron microscopic image of SELP/HA/AgNp (625 µg/mL) after a 24-hour incubation period in simulated nasal fluid.

## References

[1] Palmer J.N., Messina J.C., Biletch R., Grosel K., Mahmoud R.A. (2019). A cross-sectional, population-based survey of U.S. adults with symptoms of chronic rhinosinusitis. Allergy Asthma Proc..

[2] Fokkens W.J., Lund V.J., Hopkins C., Hellings P.W., Kern R., Reitsma S., Toppila-Salmi S., Bernal-Sprekelsen M., Mullol J., Alobid I. (2020). European Position Paper on Rhinosinusitis and Nasal Polyps 2020. Rhinology.

[3] Orlandi R.R., Kingdom T.T., Smith T.L., Bleier B., DeConde A., Luong A.U., Poetker D.M., Soler Z., Welch K.C., Wise S.K. (2021). International consensus statement on allergy and rhinology: Rhinosinusitis 2021. Int. Forum Allergy Rhinol..

[4] Kaper N.M., Aarts M.C.J., Stokroos R.J., van der Heijden G.J.M.G. (2020). Healthcare utilisation, follow-up of guidelines and practice variation on rhinosinusitis in adults: A healthcare reimbursement claims study in The Netherlands. Clin. Otolaryngol..

[5] Brandsted R., Sindwani R. (2007). Impact of depression on disease-specific symptoms and quality of life in patients with chronic rhinosinusitis. Am. J. Rhinol..

[6] Alt J.A., Mace J.C., Smith T.L., Soler Z.M. (2016). Endoscopic sinus surgery improves cognitive dysfunction in patients with chronic rhinosinusitis. Int. Forum Allergy Rhinol..

[7] Alt J.A., Smith T.L., Mace J.C., Soler Z.M. (2013). Sleep quality and disease severity in patients with chronic rhinosinusitis. Laryngoscope.

[8] Laidlaw T.M., Mullol J., Woessner K.M., Amin N., Mannent L.P. (2021). Chronic Rhinosinusitis with Nasal Polyps and Asthma. J. Allergy Clin. Immunol. Pract..

[9] Bachert C., Han J.K., Desrosiers M., Hellings P.W., Amin N., Lee S.E., Mullol J., Greos L.S., Bosso J.V., Laidlaw T.M. (2019). Efficacy and safety of dupilumab in patients with severe chronic rhinosinusitis with nasal polyps (LIBERTY NP SINUS-24 and LIBERTY NP SINUS-52): Results from two multicentre, randomised, double-blind, placebo-controlled, parallel-group phase 3 trials. Lancet.

[10] Kohanski M.A., Toskala E., Kennedy D.W. (2018). Evolution in the surgical management of chronic rhinosinusitis: Current indications and pitfalls. J. Allergy Clin. Immunol..

[11] Lourijsen E.S., Reitsma S., Vleming M., Hannink G., Adriaensen G.F.J.P., Cornet M.E., Hoven D.R., Videler W.J.M., Bretschneider J.H., Reinartz S.M. (2022). Endoscopic sinus surgery with medical therapy versus medical therapy for chronic rhinosinusitis with nasal polyps: A multicentre, randomised, controlled trial. Lancet Respir. Med..

[12] Zhang Z., Adappa N.D., Chiu A.G., Doghramji L.J., Cohen N.A., Palmer J.N. (2015). Biofilm-forming bacteria and quality of life improvement after sinus surgery. Int. Forum Allergy Rhinol..

[13] Maina I.W., Patel N.N., Cohen N.A. (2018). Understanding the Role of Biofilms and Superantigens in Chronic Rhinosinusitis. Curr. Otorhinolaryngol. Rep..

[14] Fastenberg J.H., Hsueh W.D., Mustafa A., Akbar N.A., Abuzeid W.M. (2016). Biofilms in chronic rhinosinusitis: Pathophysiology and therapeutic strategies. World J. Otorhinolaryngol. Head Neck Surg..

[15] Dlugaszewska J., Leszczynska M., Lenkowski M., Tatarska A., Pastusiak T., Szyfter W. (2016). The pathophysiological role of bacterial biofilms in chronic sinusitis. Eur. Arch. Otorhinolaryngol..

[16] Vestby L.K., Grønseth T., Simm R., Nesse L.L. (2020). Bacterial Biofilm and its Role in the Pathogenesis of Disease. Antibiotics.

[17] Foreman A., Psaltis A.J., Tan L.W., Wormald P.J. (2009). Characterization of bacterial and fungal biofilms in chronic rhinosinusitis. Am. J. Rhinol. Allergy.

[18] Bendouah Z., Barbeau J., Hamad W.A., Desrosiers M. (2006). Biofilm formation by *Staphylococcus aureus* and *Pseudomonas aeruginosa* is associated with an unfavorable evolution after surgery for chronic sinusitis and nasal polyposis. Otolaryngol. Head Neck Surg..

[19] Foreman A., Boase S., Psaltis A., Wormald P.J. (2012). Role of bacterial and fungal biofilms in chronic rhinosinusitis. Curr. Allergy Asthma Rep..

[20] Lux C.A., Wagner Mackenzie B., Johnston J., Zoing M., Biswas K., Taylor M.W., Douglas R.G. (2020). Antibiotic Treatment for Chronic Rhinosinusitis: Prescription Patterns and Associations with Patient Outcome and the Sinus Microbiota. Front. Microbiol..

[21] Zernotti M.E., Angel Villegas N., Roques Revol M., Baena-Cagnani C.E., Arce Miranda J.E., Paredes M.E., Albesa I., Paraje M.G. (2010). Evidence of bacterial biofilms in nasal polyposis. J. Investig. Allergol. Clin. Immunol..

[22] Singhal D., Psaltis A.J., Foreman A., Wormald P.J. (2010). The impact of biofilms on outcomes after endoscopic sinus surgery. Am. J. Rhinol. Allergy.

[23] Hai P.V., Lidstone C., Wallwork B. (2010). The effect of endoscopic sinus surgery on bacterial biofilms in chronic rhinosinusitis. Otolaryngol. Head Neck Surg..

[24] Smith S.S., Kim R., Douglas R. (2022). Is there a role for antibiotics in the treatment of chronic rhinosinusitis?. J. Allergy Clin. Immunol..

[25] Pynnonen M.A., Venkatraman G., Davis G.E. (2013). Macrolide therapy for chronic rhinosinusitis: A meta-analysis. Otolaryngol. Head Neck Surg..

[26] Smith A., Buchinsky F.J., Post J.C. (2011). Eradicating chronic ear, nose, and throat infections: A systematically conducted literature review of advances in biofilm treatment. Otolaryngol. Head Neck Surg..

[27] Calus L., Van Bruaene N., Bosteels C., Dejonckheere S., Van Zele T., Holtappels G., Bachert C., Gevaert P. (2019). Twelve-year follow-up study after endoscopic sinus surgery in patients with chronic rhinosinusitis with nasal polyposis. Clin. Transl. Allergy.

[28] Slavin Y.N., Asnis J., Häfeli U.O., Bach H. (2017). Metal nanoparticles: Understanding the mechanisms behind antibacterial activity. J. Nanobiotechnology.

[29] Liao C., Li Y., Tjong S.C. (2019). Bactericidal and Cytotoxic Properties of Silver Nanoparticles. Int. J. Mol. Sci..

[30] de Lacerda Coriolano D., de Souza J.B., Bueno E.V., Medeiros S.M.F.R., Cavalcanti I.D.L., Cavalcanti I.M.F. (2021). Antibacterial and antibiofilm potential of silver nanoparticles against antibiotic-sensitive and multidrug-resistant *Pseudomonas aeruginosa* strains. Braz. J. Microbiol..

[31] Sim W., Barnard R.T., Blaskovich M.A.T., Ziora Z.M. (2018). Antimicrobial Silver in Medicinal and Consumer Applications: A Patent Review of the Past Decade (2007–2017). Antibiotics.

[32] Wang L., Hu C., Shao L. (2017). The antimicrobial activity of nanoparticles: Present situation and prospects for the future. Int. J. Nanomed..

[33] Rajiv S., Drilling A., Bassiouni A., James C., Vreugde S., Wormald P.J. (2015). Topical colloidal silver as an anti-biofilm agent in a *Staphylococcus aureus* chronic rhinosinusitis sheep model. Int. Forum Allergy Rhinol..

[34] Jia M., Chen Z., Guo Y., Chen X., Zhao X. (2017). Efficacy of silk fibroin-nano silver against *Staphylococcus aureus* biofilms in a rabbit model of sinusitis. Int. J. Nanomed..

[35] Scott J.R., Krishnan R., Rotenberg B.W., Sowerby L.J. (2017). The effectiveness of topical colloidal silver in recalcitrant chronic rhinosinusitis: A randomized crossover control trial. J. Otolaryngol. Head Neck Surg..

[36] Ooi M.L., Richter K., Bennett C., Macias-Valle L., Vreugde S., Psaltis A.J., Wormald P.J. (2018). Topical Colloidal Silver for the Treatment of Recalcitrant Chronic Rhinosinusitis. Front. Microbiol..

[37] Tian D.M., Wan H.H., Chen J.R., Ye Y.B., He Y., Liu Y., Tang L.Y., He Z.Y., Liu K.Z., Gao C.J. (2022). In-situ formed elastin-based hydrogels enhance wound healing via promoting innate immune cells recruitment and angiogenesis. Mater. Today Bio.

[38] Dimatteo R., Darling N.J., Segura T. (2018). In situ forming injectable hydrogels for drug delivery and wound repair. Adv. Drug Deliv. Rev..

[39] Schilling A.L., Cannon E., Lee S.E., Wang E.W., Little S.R. (2022). Advances in controlled drug delivery to the sinonasal mucosa. Biomaterials.

[40] Schilling A.L., Kulahci Y., Moore J., Wang E.W., Lee S.E., Little S.R. (2021). A thermoresponsive hydrogel system for long-acting corticosteroid delivery into the paranasal sinuses. J. Control Release.

[41] Dandu R., Von Cresce A., Briber R., Dowell P., Cappello J., Hamidreza G. (2009). Silk–elastinlike protein polymer hydrogels: Influence of monomer sequence on physicochemical properties. Polymer.

[42] Cappello J., Crissman J., Dorman M., Mikolajczak M., Textor G., Marquet M., Ferrari F. (1990). Genetic engineering of structural protein polymers. Biotechnol. Prog..

[43] Jensen M.M., Jia W., Isaacson K.J., Schults A., Cappello J., Prestwich G.D., Oottamasathien S., Ghandehari H. (2017). Silk-elastinlike protein polymers enhance the efficacy of a therapeutic glycosaminoglycan for prophylactic treatment of radiation-induced proctitis. J. Control Release.

[44] Jensen M.M., Jia W., Schults A.J., Isaacson K.J., Steinhauff D., Green B., Zachary B., Cappello J., Ghandehari H., Oottamasathien S. (2019). Temperature-responsive silk-elastinlike protein polymer enhancement of intravesical drug delivery of a therapeutic glycosaminoglycan for treatment of interstitial cystitis/painful bladder syndrome. Biomaterials.

[45] Steinhauff D., Jensen M., Talbot M., Jia W., Isaacson K., Jedrzkiewicz J., Cappello J., Oottamasathien S., Ghandehari H. (2021). Silk-elastinlike copolymers enhance bioaccumulation of semisynthetic glycosaminoglycan ethers for prevention of radiation induced proctitis. J. Control Release.

[46] Poursaid A., Jensen M.M., Nourbakhsh I., Weisenberger M., Hellgeth J.W., Sampath S., Cappello J., Ghandehari H. (2016). Silk-Elastinlike Protein Polymer Liquid Chemoembolic for Localized Release of Doxorubicin and Sorafenib. Mol. Pharm..

[47] Griswold E., Cappello J., Ghandehari H. (2022). Silk-elastinlike protein-based hydrogels for drug delivery and embolization. Adv. Drug Deliv. Rev..

[48] Poursaid A., Price R., Tiede A., Olson E., Huo E., McGill L., Ghandehari H., Cappello J. (2015). In situ gelling silk-elastinlike protein polymer for transarterial chemoembolization. Biomaterials.

[49] Fong E., Garcia M., Woods C.M., Ooi E. (2017). Hyaluronic acid for post sinus surgery care: Systematic review and meta-analysis. J. Laryngol. Otol..

[50] Cassandro E., Chiarella G., Cavaliere M., Sequino G., Cassandro C., Prasad S.C., Scarpa A., Iemma M. (2015). Hyaluronan in the Treatment of Chronic Rhinosinusitis with Nasal Polyposis. Indian J. Otolaryngol. Head Neck Surg..

[51] Price R., Poursaid A., Cappello J., Ghandehari H. (2014). Effect of shear on physicochemical properties of matrix metalloproteinase responsive silk-elastinlike hydrogels. J. Control Release.

[52] Wiegand I., Hilpert K., Hancock R.E. (2008). Agar and broth dilution methods to determine the minimal inhibitory concentration (MIC) of antimicrobial substances. Nat. Protoc..

[53] Daly S.M., Sturge C.R., Greenberg D.E. (2017). Inhibition of Bacterial Growth by Peptide-Conjugated Morpholino Oligomers. Methods Mol. Biol..

[54] Merritt J.H., Kadouri D.E., O’Toole G.A. (2005). Growing and analyzing static biofilms. Curr. Protoc. Microbiol..

[55] Yao Y., Wang Z.C., Liu J.X., Ma J., Chen C.L., Deng Y.K., Liao B., Wang N., Wang H., Ning Q. (2017). Increased expression of TIPE2 in alternatively activated macrophages is associated with eosinophilic inflammation and disease severity in chronic rhinosinusitis with nasal polyps. Int. Forum Allergy Rhinol..

[56] Bachert C., Holtappels G. (2015). Pathophysiology of chronic rhinosinusitis, pharmaceutical therapy options. GMS Curr. Top. Otorhinolaryngol. Head Neck Surg..

[57] (2009). Biological Evaluation of Medical Devices—Part 5: Test for Invitro Cytotoxicity.

[58] Relucenti M., Familiari G., Donfrancesco O., Taurino M., Li X., Chen R., Artini M., Papa R., Selan L. (2021). Microscopy Methods for Biofilm Imaging: Focus on SEM and VP-SEM Pros and Cons. Biology.

[59] Woodworth B.A., Chandra R.K., LeBenger J.D., Ilie B., Schlosser R.J. (2009). A gelatin-thrombin matrix for hemostasis after endoscopic sinus surgery. Am. J. Otolaryngol..

[60] Surgiflo^®^ Hemostatic Matrix Essential Product Information. Ethicon, Inc. chrome-extension://efaidnbmnnnibpcajpcglclefindmkaj/https://www.jnjmedtech.com/system/files/pdf/SURGIFLO-and-Go-Sales-Brochure-135173-200320.pdf.

[61] Siu J., Klingler L., Wang Y., Hung C.T., Jeong S.H., Smith S., Tingle M.D., Wagner Mackenzie B., Biswas K., Douglas R.G. (2020). Oral antibiotics used in the treatment of chronic rhinosinusitis have limited penetration into the sinonasal mucosa: A randomized trial. Xenobiotica.

[62] Hale S.J.M., Wagner Mackenzie B., Lux C.A., Biswas K., Kim R., Douglas R.G. (2022). Topical Antibiofilm Agents With Potential Utility in the Treatment of Chronic Rhinosinusitis: A Narrative Review. Front. Pharmacol..

[63] Jensen M.M., Hatlevik Ø., Steinhauff D.D., Griswold E.D., Wei X., Isaacson K.J., Barber Z.B., Huo E., Taussky P., Jedrzkiewicz J. (2022). Protein-based polymer liquid embolics for cerebral aneurysms. Acta Biomater..

[64] Fröhlich E.E., Fröhlich E. (2016). Cytotoxicity of Nanoparticles Contained in Food on Intestinal Cells and the Gut Microbiota. Int. J. Mol. Sci..

[65] Wu L., Luo Y. (2021). Bacterial Quorum-Sensing Systems and Their Role in Intestinal Bacteria-Host Crosstalk. Front. Microbiol..

[66] Singh B.R., Singh B.N., Singh A., Khan W., Naqvi A.H., Singh H.B. (2015). Mycofabricated biosilver nanoparticles interrupt *Pseudomonas aeruginosa* quorum sensing systems. Sci. Rep..

[67] Haidari H., Kopecki Z., Bright R., Cowin A.J., Garg S., Goswami N., Vasilev K. (2020). Ultrasmall AgNP-Impregnated Biocompatible Hydrogel with Highly Effective Biofilm Elimination Properties. ACS Appl. Mater. Interfaces.

[68] Liu T., Aman A., Ainiwaer M., Ding L., Zhang F., Hu Q., Song Y., Ni Y., Tang X. (2021). Evaluation of the anti-biofilm effect of poloxamer-based thermoreversible gel of silver nanoparticles as a potential medication for root canal therapy. Sci. Rep..

[69] Loo C.Y., Young P.M., Lee W.H., Cavaliere R., Whitchurch C.B., Rohanizadeh R. (2014). Non-cytotoxic silver nanoparticle-polyvinyl alcohol hydrogels with anti-biofilm activity: Designed as coatings for endotracheal tube materials. Biofouling.

[70] Gurina D., Surov O., Voronova M., Zakharov A., Kiselev M. (2019). Water Effects on Molecular Adsorption of Poly(N-vinyl-2-pyrrolidone) on Cellulose Nanocrystals Surfaces: Molecular Dynamics Simulations. Materials.

[71] Maslova E., Eisaiankhongi L., Sjöberg F., McCarthy R.R. (2021). Burns and biofilms: Priority pathogens and in vivo models. NPJ Biofilms Microbiomes.

