# Supplementary material for: Matrix-Mediated Delivery of Silver Nanoparticles for Prevention of Staphylococcus aureus and Pseudomonas aeruginosa Biofilm Formation in Chronic Rhinosinusitis
Source: Pharmaceutics. 2023 Oct 5;15(10):2426. doi: 10.3390/pharmaceutics15102426 (PMC10610389; doi:10.3390/pharmaceutics15102426)
Supplement: Supplementary file 1 [file pharmaceutics-15-02426-s001.zip › pharmaceutics-2615334-supplementary.pdf]

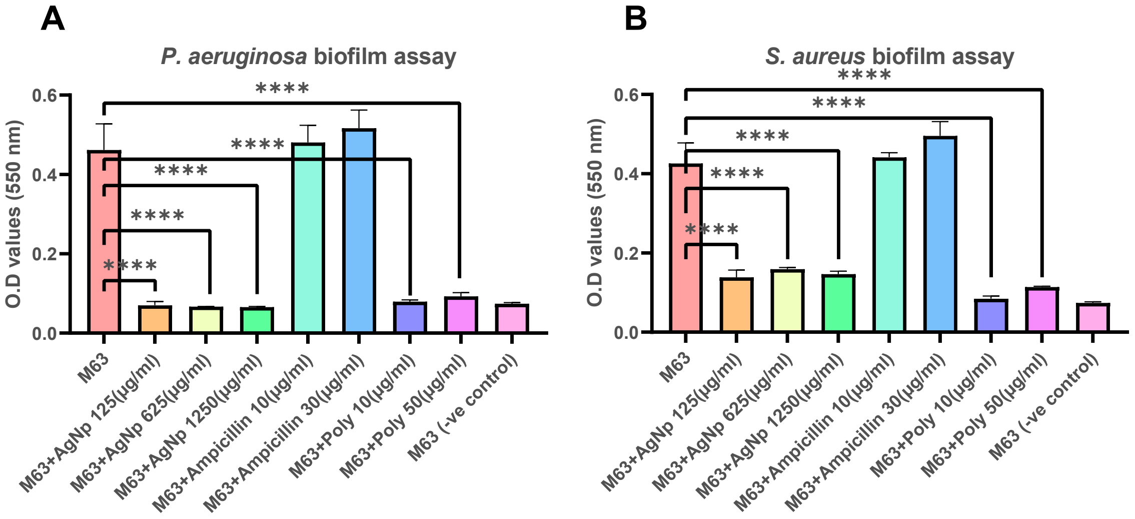

Figure S1. Biofilm disruption assessments with silver nanoparticles (AgNp) and antibiotic controls using the crystal violet assay. AgNp prevent biofilm-forming properties of A) *Pseudomonas aeruginosa*, B) *Staphylococcus aureus* in minimal growth medium (M63). N=3. Data are represented as the mean ± SD. ****$p < 0.0001$. Polymyxin B (poly).

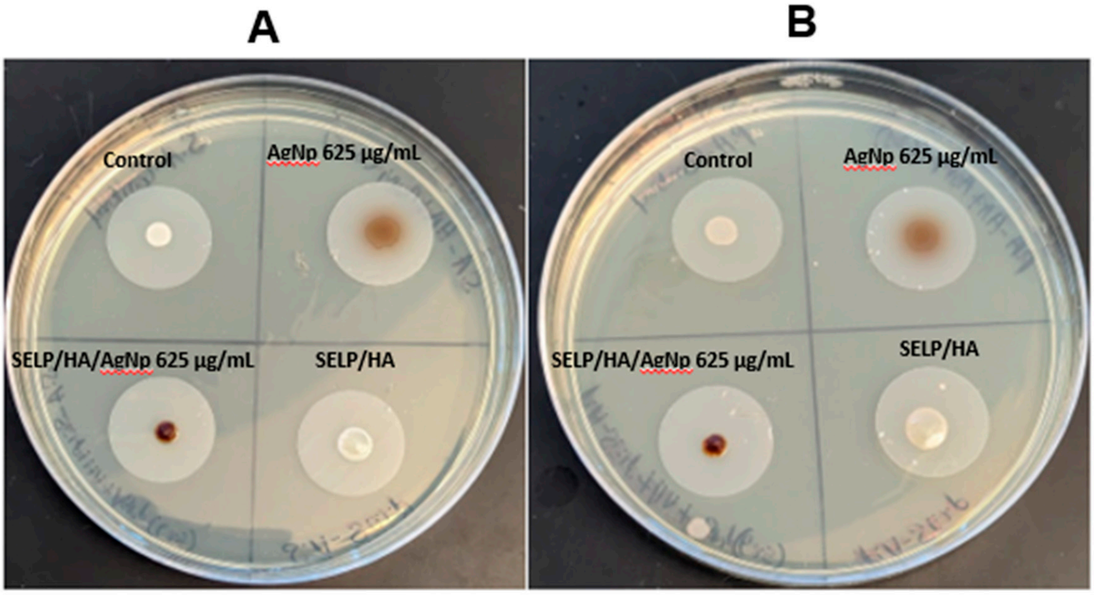

Figure S2. Agar plates showing the inhibition of bacterial growth by silk-elastinlike protein polymer (SELP) (11%) with HA (0.2 %) and AgNp 625 µg/ml (SELP/HA/AgNp 625 µg/ml) and AgNp (625 µg/ml) formulations after a 24-hour incubation period with A) *Staphylococcus aureus* and B) *Pseudomonas aeruginosa*.

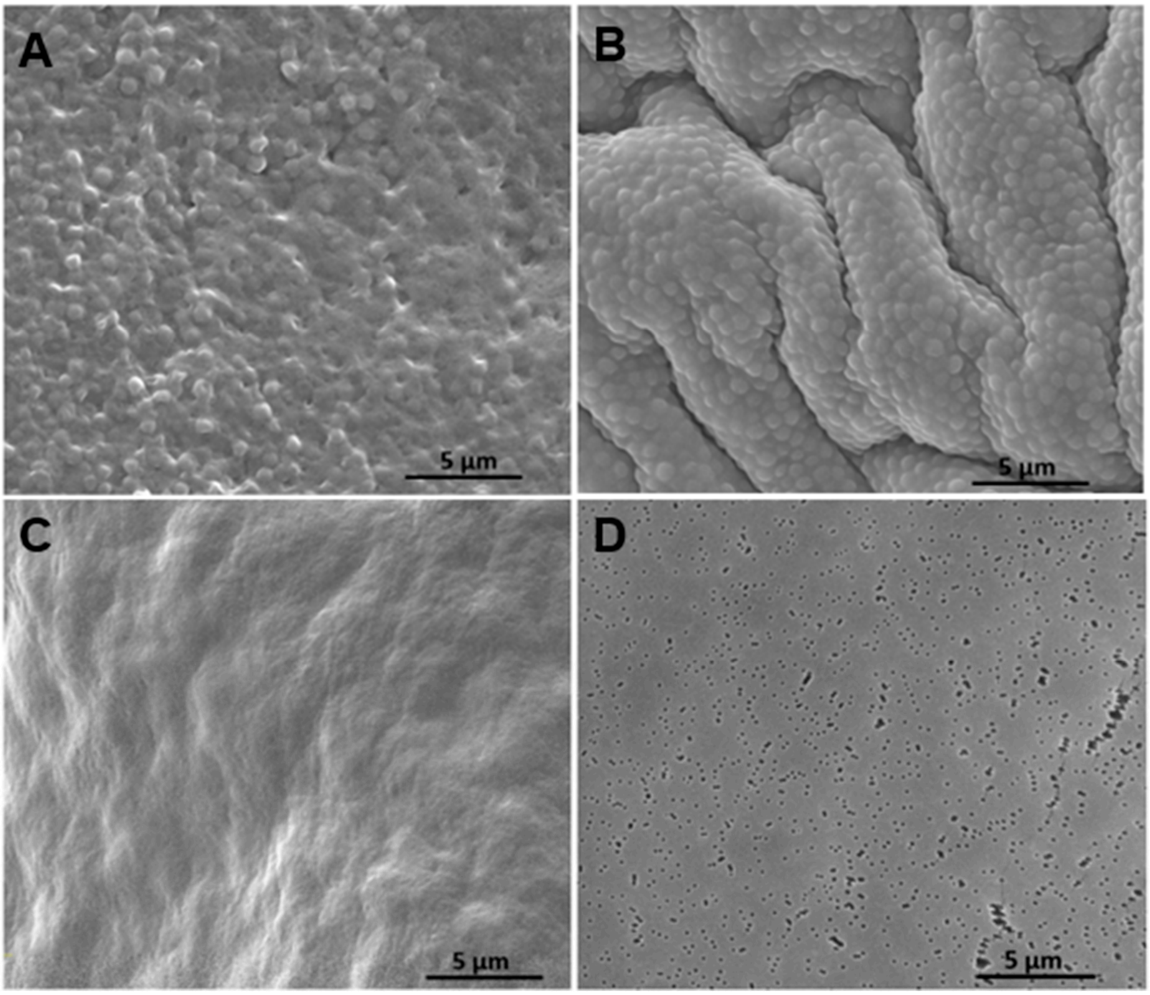

Figure S3. *Staphylococcus aureus* colony biofilm assessments after treatment with different antibacterial formulations using scanning electron microscopy (SEM). Images demonstrate the presence of biofilms generated on polycarbonate filters A) without treatment and B) with SELP/HA treatment (controls). Treatment with C) SELP/HA/AgNp and D) ciprofloxacin control result in complete biofilm prevention after a 24-hour incubation period. Silk-elastinlike protein polymer (SELP), hyaluronic acid (HA), silver nanoparticles (AgNp).

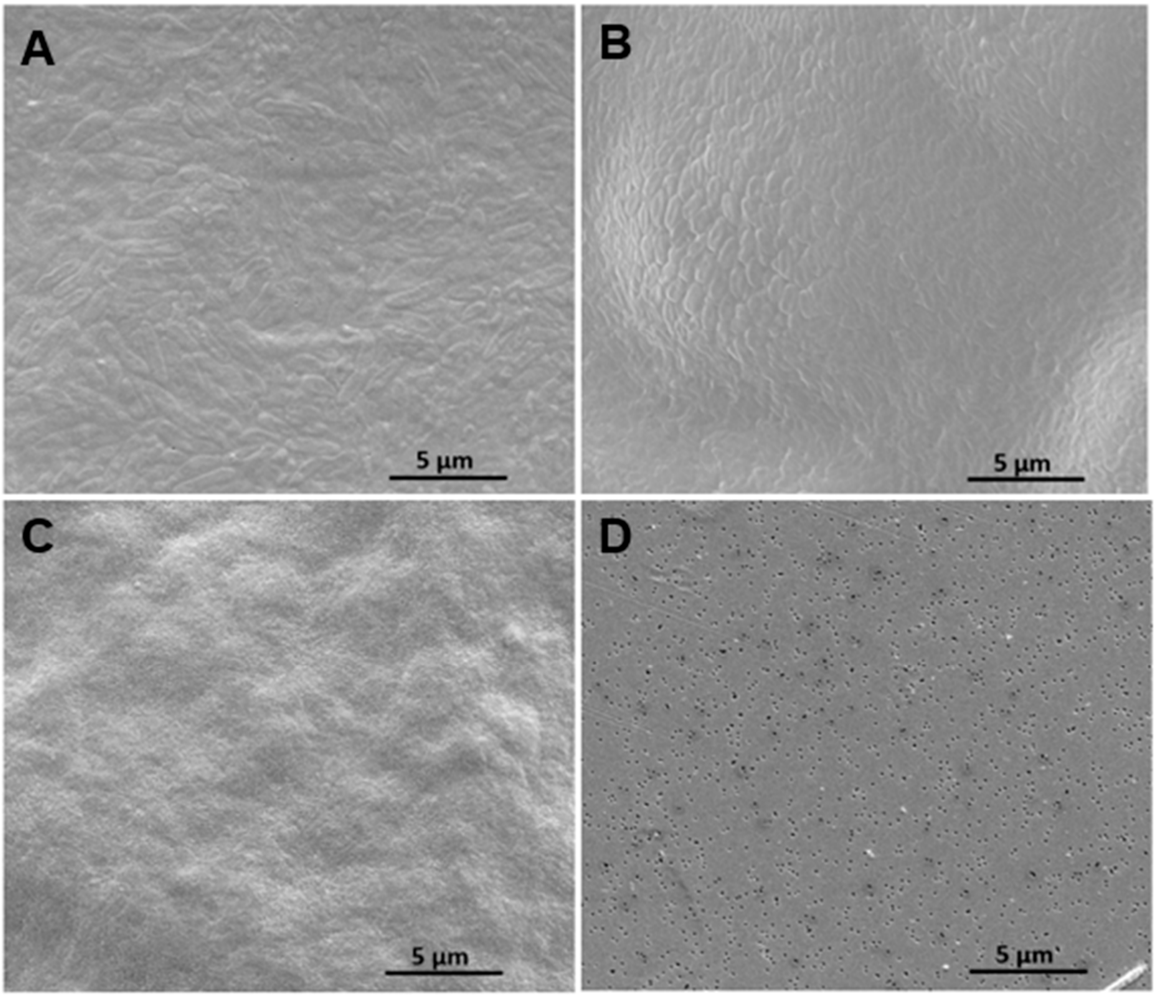

Figure S4. *Pseudomonas aeruginosa* colony biofilm assessments after treatment with different antibacterial formulations using scanning electron microscopy (SEM). Images demonstrate the presence of biofilms generated on polycarbonate filters A) without treatment and B) with SELP/HA treatment (controls). Treatment with C) SELP/HA/AgNp and D) ciprofloxacin control result in complete biofilm prevention after a 24-hour incubation period. Silk-elastinlike protein polymer (SELP), hyaluronic acid (HA), silver nanoparticles (AgNp).

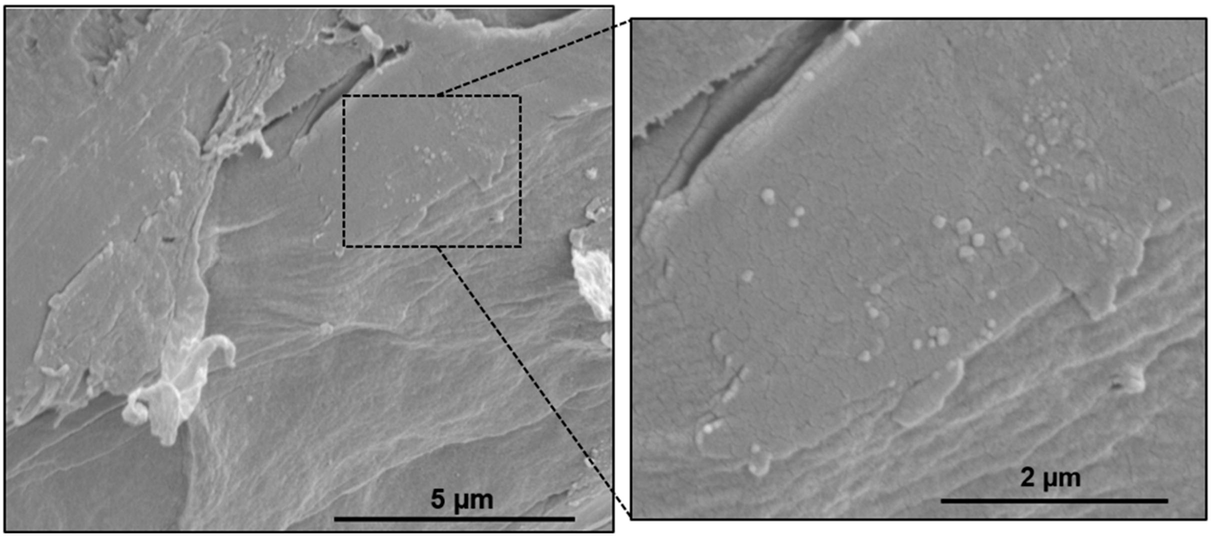

Figure S5. Scanning electron microscopic image of SELP/HA/AgNp (625 μg/mL) after a 24-hour incubation period in simulated nasal fluid. AgNp interacts with SELP-815K, leading to sustained release of Ag^{+} ions. Silk-elastinlike protein polymer (SELP), hyaluronic acid (HA), silver nanoparticles (AgNp).
